# The scientific basis of vascularized bone grafts: Models and perfusion assessments, a systematic review^✰^

**DOI:** 10.1016/j.jpra.2026.01.034

**Published:** 2026-01-30

**Authors:** Marcus Wölffer, Radu Olariu, Alex Woollard, Cédric Zubler

**Affiliations:** a Department of Plastic and Hand Surgery, Inselspital University Hospital Bern, University of Bern, Freiburgstrasse 18, Bern, Switzerland; b Department of Plastic Surgery, Royal Free Hospital, Hampstead, London, United Kingdom; c Department of Plastic and Reconstructive Surgery, Great Ormond Street Hospital for Children, Guilford St, London, United Kingdom

**Keywords:** Vascularized bone graft, Bone flap, Perfusion assessment, Anatomical cadaver and animal models, Orthoplastic

## Abstract

Vascularized bone grafts (VBGs) are assumed to have advantages over non-VBGs, including preservation of cell viability and accelerated osteogenesis. Indications include the reconstruction of large bone defects, poor vascularity and previous radiation. However, clinical evidence supporting their superiority is limited. A return to the basic science of animal and cadaver models seems appropriate. This systematic review focuses on perfusion assessment in studies on VBGs. Only by understanding the vascular anatomy and ensuring bone perfusion is truly maintained in VBGs, can we expect to find differences to non-VBGs in subsequent experiments.

A systematic review was performed according to the PRISMA checklist. In February 2025, MEDLINE via PubMed, Embase, and the Cochrane Library databases were systematically searched for studies presenting animal or cadaver anatomical models of VBGs. Exclusion criteria were: language other than English, missing full-text publication, systematic reviews, prefabrication and tissue engineering, anatomical descriptions of bone grafts without perfusion assessments. Inclusion criteria were: original research on VBGs in animals or cadavers assessed by contrast agents, bone graft perfusion models. Out of 520 initially identified publications, 25 studies were included. Disagreements on the eligibility of articles were resolved by discussion with a senior author.

Pedicled and free VBGs from various anatomical sites were examined, covering indications, contraindications, benefits, and limitations. Several perfusion assessment techniques were employed, including histology, CT, MRI, technetium-based scintigraphy, near-infrared fluorescence imaging (NIRFI), angiography, radioactive microspheres, and fluorochrome bone labeling (FBL). Histology helps confirm viable osteocytes, while FBL and conventional as well as CT-angiography provide insight into vascular integrity. Meanwhile, scintigraphy and MRI offer indirect perfusion markers but demonstrate high false-positive rates. NIRFI seems accurate intraoperatively but is of limited use postoperatively. Advantages and disadvantages of each method are explored.

Reliable perfusion assessment is crucial in research on VBGs and requires a multimodal approach. Future research should standardize models and timing of perfusion assessment while accounting for confounding factors such as spontaneous neoangiogenesis and considering pitfalls of each modality.

## Introduction

Relevant bone defects can result from oncologic resections, infections, trauma or congenital conditions and pose a significant challenge in orthoplastic surgery.^1^ Multiple techniques have been described to address these defects, such as bone transport,^2^^,^^3^ the Masquelet-technique^4^ or non-vascularized and vascularized bone grafts (VBGs).^5^ While expertise and preferences differ between centers, VBGs or non-VBGs are most commonly used. Traditionally, VBGs have been recommended over non-VBGs for the reconstruction of bone defects measuring more than 6 cm, however, no clear evidence for this recommendation has been identified yet.^6^ Other indications for VBGs include recipient site necrosis and inadequate vascularity of local tissues,^7^ prior radiation with areas of relative avascularity or lack of soft tissues,^8^ and difficult fractures or non-unions.^9^ VBGs are often used in head and neck reconstructions with their most common application being in oromandibular reconstruction after oncologic resections or osteoradionecrosis.^1^^,^^10^ They have been shown to resist infection by providing blood supply^11^ and bone healing was reported to be accomplished in a shorter period of time due to primary healing with preserved osteocytes.^12^ The preference accorded to VBGs stems from the idea that the continuous blood supply ensures a preservation of biological characteristics of the graft. VBGs undergo hemodynamic and histological changes that are different from the “creeping substitution” described by Phemister to indicate the resorption, infiltrative revascularization and replacement of necrotic bone by recipient osteoblasts for non-VBGs.^13^ However, these processes are still insufficiently understood.^14^

The increasing success of orthoplastic surgery for limb salvage has made addressing large bony defects also a priority in extremity reconstruction. Microsurgical techniques allow the transfer of sizable bone grafts along with their intrinsic vasculature.^15^ When Taylor et al. performed the first vascularized free fibula flap in 1975^5^, the limitations in reach of pedicle-bound grafts were lifted, expanding applicability of VBGs. Nowadays, many VBG donor sites are available, including the dorsal and volar distal radius, proximal radius, humerus, fibula, iliac crest, medial and lateral femur condyle, scapula and ribs.^16^^,^^17^

However, meta-analyses have shown little evidence to prove the assumed superiority of VBGs over non-VBGs.^18^^,^^19^ While VBGs offer advantages in the above-mentioned cases, they are technically more challenging, require longer operation times and risk higher donor site morbidity.^6^ Their availability is limited and size and shape mismatches may lead surgeons to choose other options. While some systematic reviews have compared union rates of VBGs and non-VBGs, there is also a lack of direct comparisons between different types of VBGs including indications, advantages and outcomes.^20^ Furthermore, clinical studies comparing VBGs and non-VBGs, controlling for potential biases such as indication, defect size or vascularity of local tissues, may not be feasible for ethical reasons.

A return to the basic science and knowledge established in animal or cadaver models seems appropriate to gain a better understanding about this missing data. In this article we aim to review existing experimental VBGs models with a focus on how the perfusion was assessed. Only by understanding the vascular anatomy and ensuring blood flow is maintained in the grafts, can we expect to find any true difference to non-VBG comparison groups in subsequent experiments and eventually clinical practice.

## Methods

This systematic review was conducted according to the PRISMA-2020-checklist.^21^ No ethical approval was required. The study was not registered.

### Search strategy and selection criteria

Data collection was performed according to the principles of the Cochrane Collaboration.^22^ Keyword selection was based on the PICO model.^23^ In February 2025, MEDLINE via PubMed, Embase, and the Cochrane Library databases were systematically searched for studies presenting animal or cadaver anatomical models of VBGs. The search terms used described the study population and intervention: ((vascularized bone graft*) OR (vascularized bone flap*) OR (vascularized bone transplant*) OR (vascularized bone graft*) OR (vascularized bone flap*) OR (vascularized bone transplant*)) AND ((model*) OR (animal*) OR (cadaver*)). Exclusion criteria were: language other than English, missing full-text publication, systematic reviews, prefabrication and tissue engineering, anatomical descriptions of bone grafts without perfusion assessments. Inclusion criteria were: original research on VBGs in animals or cadavers assessed by contrast agents, bone graft perfusion models. Two reviewers (MW and CZ) independently performed full-text screening. Any disagreements on the eligibility of articles were resolved by discussion with a senior author (RO). A cross-check of the references from the original studies was performed to identify potential additional articles.

### Data extraction

Two reviewers (MW, CZ) performed data extraction. The following baseline characteristics were extracted from the included studies: first author, year of publication, type of specimens (cadavers or animals) including sample size and type of animal, type of VBG, blood supply, type of contrast agent and perfusion assessment, advantages, disadvantages, indications and contraindications. Results were entered into a Microsoft 365 Excel spreadsheet, which was used to organize data during analyses.

The primary outcomes of interest were the anatomical basis, perfusion assessment methods and results of VBG models.

## Results

### Literature search

**Figure 1:**
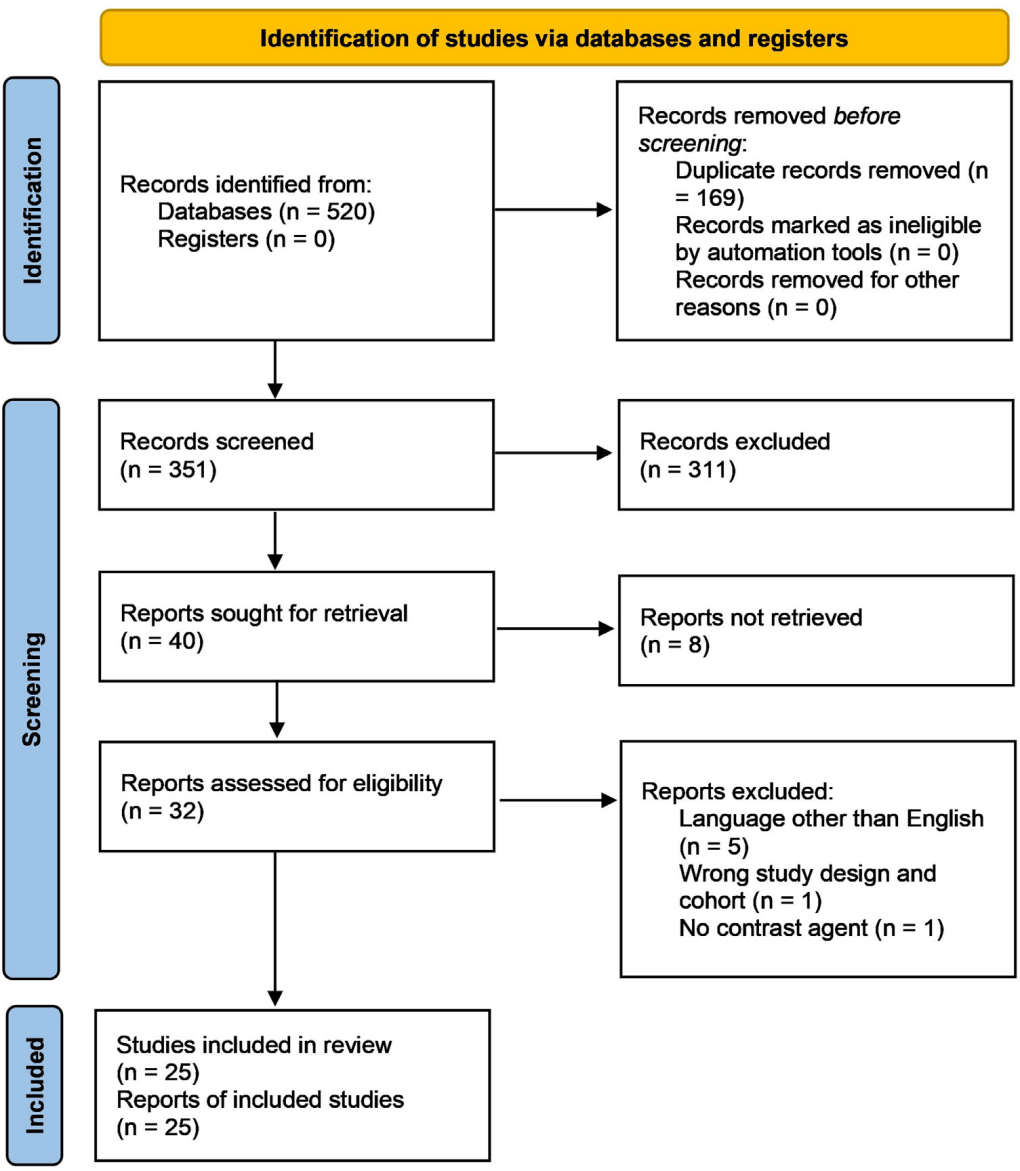
PRISMA 2020 flow diagram on the database search.

About 520 publications were initially identified. 169 duplicate records were removed. The remaining 351 records were subjected to title and abstract screening by two reviewers independently (MW, CZ). Of these, 311 records were excluded, and 40 reports included in the secondary review. These records included the main categories of cadaver and animal anatomical models as well as perfusion assessment records. 32 studies were assessed for eligibility and 25 included in the review. The search syntax is demonstrated in Figure 1.

### Models

Table 1 shows the anatomical VBGs models described in cadaver or animal studies. A predominance of cadaver rather than animal studies can be noted. Sample sizes range from 5 to 104. About 4 studies use free VBGs, 9 use pedicled VBGs. 5 studies use VBGs based on different regions of the radius. All studies use contrast agents to visualize the vascular network, with 7 studies using latex. Discussed advantages and disadvantages revolve around the requirement for VBGs not to create donor defects, to have an optimal perfusion with a long pedicle, to require a short and simple operative procedure with good anatomical accessibility, and to have a sufficient size and bone structure for the intended reconstruction. The typical indications proposed include non-union, chronic osteomyelitis, midface or mandible reconstruction and avascular necrosis. While most studies do not mention specific contraindications, previous local surgery is highlighted by some.

**Table 1:** Results of cadaver or animal studies describing anatomical models of VBGs.

Study	Year of study	Specimens	Sample size	Type of animal	Pedicled or free	Type of bone graft	Arterial supply	Type of contrast agent	Indication	Contraindication	Advantages	Disadvantages
Pierer et al.^43^	1992	Cadavers	104	Human	Pedicled	Second metacarpal	Branches of the radial artery or the palmar metacarpal artery	Liquid glue, radiopaque minimum dye, methylene hydrocyanic acid	Partial graft for bone reconstruction, complicated fractures, non-unions, arthrodesis of small joints, avascular bone necrosis. Total graft for thumb reconstruction in cases of emergency metacarpal amputation		Size and bone structure match the thumb	
Sundine et al.^8^	2000	Cadavers	25	Human	Free	Inferior scapula angle	Descending osseous branch of the circumflex scapular artery and transverse branch of the thoracodorsal artery	Microfil	Massive midface defects after oncologic resection or gunshot wounds		Only a thin muscle sheet kept in continuity with the bone to protect periosteal branches, allows independently oriented segments, flat bone suitable for palate or orbital floor reconstruction	
Mathoulin et al.^44^	2004	Cadavers	40	Human	Pedicled	Palmar distal radius	Radial palmar carpal artery	Latex	Scaphoid non-union			
Waitayawinyu et al.^45^	2008	Cadavers	23	Human	Pedicled	Distal radius	1,2 ICSRA	Red latex	Scaphoid non-union	Styloid fracture		Difficulty to visualize vessels, mobilization might be impossible without disrupting the dorsal scaphoid branch
Pagnotta et al.^46^	2012	Cadavers	5	Human	Pedicled	Dorsal distal radius	Posterior interosseous artery	Latex	Diaphysis ulnar nonunion with bone defects of up to 2cm		Limited to the forearm without further donor site morbidity	Small size of graft (2–3 cm maximum length)
Moor et al.^47^	2012	Cadavers	30	Human	Pedicled	Acromion	Acromial branch of the thoracoacromial trunk	Angiofil, blue resin	Young patients with avascular necrosis of the humeral head or atrophic non-union of the clavicle	Previous shoulder surgery	Accessibility, limited morbidity, wedge profile	Limited bone wedge due to necessary deltoid refixation, CAL must be preserved at the coracoid to protect arterioles
Bermel et al.^48^	2014	Cadavers	16	Human	Pedicled	Dorsal base of the second metacarpal	second dorsal metacarpal artery	India ink, gelatin, mannitol	Kienböck’s disease		Limited donor-site morbidity as the bone graft is taken out of the immobile unit of the second and third metacarpals, trapezoid and capitate	Possible restricted index mobility, carpometacarpal arthritis, limited size for reconstruction of the proximal scaphoid
Wong et al.^49^	2015	Cadavers	31	Human	Free	Lateral femoral condyle	Superolateral geniculate artery	Pink or blue latex			Short operation, large areas of bone can be tailored to numerous shapes, no major vessel harvested, chimeric designs with skin paddle, soft tissue, bone or cartilage	Shorter pedicle and smaller domain of periosteal perfusion than the medial femoral condyle graft
Varma et al.^7^	2020	Cadavers	22	Human	Pedicled	Distal radius	1,2 ICSRA	Polyvinyl chloride, acetone, red resin	Scaphoid non-union			Delicate dissection, dye visualization is necessary to locate perforators, harvesting the area with the highest density of perforators is necessary
Yang et al.^50^	2020	Cadavers	13	Human	Free	Cortico-cancellous olecranon	Perforators of the posterior ulnar recurrent artery	Blue latex, india ink			Proximity to upper extremity operation sites, ease of harvest, avoidance of neurovascular structures, hidden scar, few complications	Postoperative immobilization, pain and weakness in wrist flexion, release of the ulnar nerve, injury to the medial antebrachial cutaneous nerve, size limited to 3 cm x 2 cm x 2cm
Barrera-Ochoa et al.^51^	2025	Cadavers	10	Human	Pedicled	Proximal radius	Radial artery	Latex	Elbow defect (>6 cm) with chronic osteomyelitis and inadequate perfusion, where allografts or arthoplasties are contraindicated	Previous forearm surgery or trauma, inadequate hand perfusion	Proximity to the defect, regional anesthesia	Complications such as sacrifice of the radial artery, injury to the ulnar artery and ulnar, radial and median nerves, distal radius impingement
Chen et al.^9^	2000	Animals	12	Rats	Free	Fibula	Popliteal vessels	Lead oxide latex	Long bone defects, pseudarthrosis of the tibia or forearm, osteomyelitis, mandible reconstruction		Bone length and straight, thick cortex	
Giessler et al.^12^	2008	Animals	10	Rabbits	Pedicled	Femoral diaphysis	Nutrient vessel from the lateral femoral circumflex artery	Microfil	Long bone reconstruction			Free graft: anastomoses include the lateral femoral origin, femoral vessels are ligated distal to the origin of the deep femoral vessels

1,2 ICSRA, 1,2 intercompartmental supraretinacular artery; CAL, Coracoacromial ligament.

### Perfusion

Table 2 shows studies assessing perfusion in VBGs. 12 out of 14 studies were conducted in animals with 7 out of these in dogs. Sample sizes range from 3 to 65. 8 studies use free VBGs. Most studies use a combination of different techniques to assess perfusion. These include histology (*n* = 9), radiography (*n* = 3), computed tomography (CT; *n* = 3), magnetic resonance imaging (MRI; *n* = 1), technetium-based scintigraphy (*n* = 2), indocyanine green-based near-infrared fluorescence imaging (ICG-based NIRFI; *n* = 1), perfusing stain with methylene blue (*n* = 1), different types of angiography (*n* = 4), radioactive microspheres (*n* = 3) and fluorochrome bone labeling (*n* = 6).

**Table 2:** Studies assessing perfusion in VBGs.

Study	Year of study	Specimens	Sample size	Type of animal	Pedicled or free	Type of bone graft	Arterial supply	Type of perfusion assessment
Wong et al.^49^	2015	Cadavers	31	Human	Free	Lateral femoral condyle	Superolateral geniculate artery	Fluoroscopic angiography
Davenport et al.^20^	2024	Cadavers	7	Human	Pedicled	3 different regions of distal radius	1,2 ICSRA, 4 ECA, volar carpal artery	Nano-CT with barium
Berggren et al.^24^	1982	Animals	9	Dogs	Free	Posterior rib, posterolateral segmental rib	Nutrient vessel with medullary and periosteal perfusion, or periosteal vessels alone based on the internal mammary artery	Histology, fluorochrome bone labeling with intravenous oxytetracycline (500 mg), alizarin complexone (30mg/kg) and DCAF (20mg/kg), microangiography with Colorpaque, Tc-based scintigraphy
Teissier et al.^14^	1985	Animals	32	Rabbits	Free	Fibula	Posterior tibial artery	Histology, Ce-labeled microspheres
Shi et al.^25^	1986	Animals	31	Rabbits and dogs	Free	Distal radius, anterointernal tibia, anterior intermuscular septum and fibula	Fasciosteal vessels	Histology, fluorochrome bone labeling with tetracycline, perfusing stain with methylene blue
Weiss et al.^30^	1988	Animals	65	Rats	Free	Knee allograft	Femoral vessels	Histology, histomorphometry, fluorochrome bone labeling with subcutaneous DCAF (20mg/kg) and subcutaneous alizarin complexone (30mg/kg)
Gonzales del Pino et al.^28^	1990	Animals	15	Dogs	Free	Rib	Posterior intercostal artery	High-resolution CT with micronized barium sulphate
Canosa et al.^15^	1994	Animals	18	Dogs	Free	Anterior segmental rib	Posterior intercostal vessels and periosteal circulation	Radiography, angiography, Tc-based scintigraphy, histology, CT
Gur et al.^26^	1999	Animals	8	Pigs	Pedicled	Osteotomized fibula	Caudal tibial artery	Radiography, fluorochrome bone labeling with subcutaneous xylenol orange (90mg/kg), intravenous tetracycline (30mg/kg) and subcutaneous alizarine (30mg/kg), histology
Tu et al.^29^	2000	Animals	16	Dogs	Pedicled	Reverse flow dorsal and palmar or solely dorsal radius	2,3 ICSRA, 3,4 ICA	Radiolabeled microspheres
Kobayashi et al.^10^	2005	Animals	3	Dogs	Pedicled	Tibia	Caudal tibial artery	Histology, fluorochrome bone labeling with oxytetracycline
Giessler et al.^12^	2008	Animals	10	Rabbits	Pedicled	Femoral diaphysis	Nutrient vessel from the lateral femoral circumflex artery	Microangiography with Microfil, histology
Willems et al.^27^	2011	Animals	4	Dogs	Pedicled	Reverse-flow dorsal radial distal radius	2,3 ICSRA and 3,4 ICA	Ce-labeled microspheres, MRI, radiography, histology, fluorochrome bone labeling and fluorescence microscopy
Nguyen et al.^1^	2012	Animals	16	Pigs	Free	Radial forelimb osteomyocutaneous, hindlimb fibula	Brachial artery	ICG-based NIRFI, clinical observation, Doppler

ICG-based NIRFI, indocyanine green-based near-infrared fluorescence imaging; CT, computed tomography; MRI, magnetic resonance imaging; Tc, technetium; Ce, cerium; ICSRA, intercompartmental supraretinacular artery; ECA, extensor compartmental artery; ICA, intercompartmental artery.

#### Histology

While normally stained osteocytes and contrast-filled vessels are signs of viable grafts,^24^ marrow degeneration and unviable osteocytes are signs of non-viability.^25^ In one study an initial phase of 5 weeks with zones of cortical empty lacunae and creeping substitution despite patent anastomoses is described, with viability increasing and being completed at 14 weeks. The medullary cavity shows initial cell viability in only two thirds of its surface, but total viability and new trabecular bone formation are completed at 8 and 14 weeks.^15^ Other authors found completely viable cells and new bone formation at 3 weeks already.^26^

#### Radiography

Bone volume, hypertrophy, height and width assessed by radiography have been used as indirect markers of viability and therefore perfusion; however, results are inconsistent. While some authors found that bone volume did not change with time with equal height-to-width ratios,^27^ others found less than 20% hypertrophy at 5 weeks and progressive increase of graft width thereafter.^15^ Another study described marked hypertrophy and increased transverse width without differences in length.^26^

#### Computed tomography

Barium-based CT was used to confirm patency of periosteal and medullary vessels.^15^ In high-resolution CT periosteal proliferation was noted at 8 weeks and at 12 weeks anastomoses to medullary vessels crossed the graft interface.^28^ Nano-CT was able to quantify the vascular volume of different distal radius VBGs including the number of cortical perforators.^20^

#### Magnetic resonance imaging

MRI showed decreased T1 and increased T2 signals, representing early revascularization. However, these results did not correlate with flow measurements.^27^

#### Technetium-based scintigraphy

A relative increase in uptake at the grafting site was noted in 88.9% of animals.^15^ Normal or increased uptake in all specimens was found in a rib graft model.^24^

#### Near-Infrared fluorescence imaging

ICG-based NIRFI was used to identify perfusion directly after flap harvest, demonstrating blood flow in the vascular pedicle and the osteotomy sites. No fluorescence was seen in pedicles clamped before administration of ICG or devascularized control flaps.^1^

#### Angiography

Angiography successfully identified proliferating periosteal vessels and identified occluded vessels in 37.5% of normal technetium (Tc)-based scintigraphy evaluations proving the latter method’s high false positive rate.^15^ Microangiography showed normal vascular architecture in all specimens^24^ and patency in all pedicles after 16 weeks with intraosseous vessel density comparable to control femora.^12^

#### Radioactive microspheres

Cerium-141-labeled microspheres showed a substantially higher blood flow in VBGs when compared to controls.^27^ More precisely, after an initial hypervascularization of 3 weeks, a decrease between days 30 to 90, and stabilization at the level of untreated controls between the third and fifth months was found.^14^ Other authors found this hypervascularization to occur only after 2 weeks, and noted a flow decrease of nearly 50% immediately after elevation when compared to untreated controls.^29^

#### Fluorochrome bone labeling

Different fluorochromes can be administered starting directly after grafting and carried on until sacrifice. Successful administration and histological evidence of these in the VBGs have been reported between 75%^24^ and 100%.^27^ Labeling has been successful in the cortex, trabeculae and periosteum.^25^ Oxytetracycline administered directly after grafting showed high vascularity and was observed up to 12 weeks postoperatively.^10^ Subperiosteal and endosteal deposition of alizarin complexone given 48 h before sacrifice suggested intact perfusion in a fibula model. Previously given xylenol orange or tetracycline were found only endosteally, explained by rapid bone turnover with periosteal washout.^26^ Presence of 2,4-bis [N,N’-di(carbomethyl)aminomethyl] fluorescein (DCAF) given postoperatively and absence of alizarin complexone administered before sacrifice indicate an initially patent anastomosis that became occluded.^30^

## Discussion

### Models and latex perfusion

Surgical techniques differed according to the graft used, and an in-depth discussion of these would go beyond the scope of this review. However, most studies establishing models used latex to visualize vascularization and facilitate dissection. Latex is injected into vessels of the region of interest. Pure injections serve as visual guide during dissection, while radiopaque solutions are mixed with barium sulfate. They allow for biplanar fluoroscopy, guiding injections and surgical planning, or CT scans visualizing vessels in 3D reconstructions.^31^

Latex preserves the relationships between vessels and surrounding tissue, which is valuable in anatomical comparative studies. Sufficient perfusion is achieved when back-flow through contralateral arteries is observed. The radiopaque approach allows tracking vascular flow patterns. Its density and adherence to vessel walls provide excellent contrast in imaging.^31^

Yet, over-perfusion can lead to vessel ruptures with leakage. Pure latex injections are non-standardized and present inconsistent results. A successful application depends on the preservation state, anatomy and vessel size. Radiopaque latex is too viscous to perfuse the capillary bed.^31^ The technique therefore seems fit for exploration of the vascular macro-anatomy but does not provide any dynamic or microvascular information.

### Characteristics of VBGs

Preservation of perfusion results in VBGs remaining viable without creeping substitution happening,^32^ while revascularization after free VBG transfer has been shown to initiate bone turnover.^27^ Normally, bridging by new bone begins at 4 weeks and the external cortical layer is continuous at 12 weeks. Local blood flow remains markedly higher even after 1 year.^10^ The majority of segments in an osteotomized fibula model have been shown to heal without callus formation and only minimal fibrosis.^26^ The accelerated osteogenesis seen in VBGs has been explained by the transplantation of viable marrow cells, increased nutrients and osteoprogenitor cells.^27^ Other authors found early bone necrosis in VBGs despite patent anastomoses,^15^ which might be explained by insufficient perfusion after harvest. The initial but not immediate phase of hypervascularization detected by several authors was attributed to a reaction to ischemia during the adaptive phase of vasculature to new hemodynamic conditions, the sympathectomy achieved by skeletonizing the pedicle, or the inflammatory response.^14^^,^^15^

### Periosteal and medullary perfusion

Newly formed bone has been shown to be greater in proximal areas of VBGs, suggesting a decreased osteogenesis due to reduced perfusion distally. The same was suggested by bone labeling.^10^ In normal bone, periosteal vessels play a minor role in cortical perfusion. Blood flows centrifugally from medullary to cortical vessels, due to the physiological high-pressure in the medullary system. In ischemic situations, this may be reversed to assure bone survival. Either periosteal or medullary systems alone may be capable of sustaining a sufficient perfusion. Eventually, most VBGs were based solely on periosteal perfusion because of its ease of harvest.^24^ Other authors confirmed this reliance on periosteal circulation with periosteal washout of fluorochromes given early.^26^ VBGs supplied by periosteal vessels establish a circulation to medullary vessels and have shown similar viability compared to grafts supplied by medullary and periosteal vessels together.^24^

### Methods of perfusion assessment

**Figure 2:**
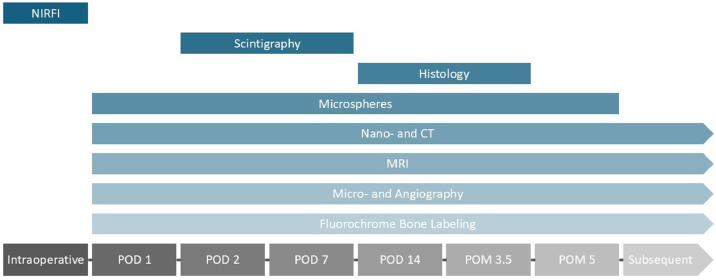
Recommended perfusion assessment techniques over time. POD, postoperative day; POM, postoperative month; NIRFI, Near-infrared fluorescence imaging.

No single technique has been established to reliably assess and monitor viability of VBGs.^1^ Postoperatively, skin islands are often used to monitor anastomotic patency, but do not guarantee osseous perfusion. Table 3 provides an overview of perfusion assessment methods with respective advantages, disadvantages and recommended use. Figure 2 provides an overview of the perfusion assessment techniques in regards to the recommended timing of use. Additionally, Table 4 provides an overview of recommended perfusion assessment methods according to the timeline and intended clinical or experimental use. In a clinical setting, destructive or ex vivo assessment methods, i.e., histology, fluorochrome bone labeling, nano-CT, microangiography, are not adequate. Intraoperative evaluation with NIRFI and postoperative monitoring with radioactive microspheres might seem to be the most adequate and reliable combination of methods in this setting. However, different factors including cost considerations, availability and timing will influence this decision.

**Table 3:** Methods of perfusion assessment with respective advantages, disadvantages and recommended use.

Type of perfusion assessment	Advantages	Disadvantages	Recommended use
Histology	Reliable correlation with blood flow, spatial resolution, functional information	Time and personnel-intensive, destructive, incomplete 3D visualization, falsifications due to preparation techniques	Monitoring ex vivo
Nano-CT	High resolution, non-destructive, 3D, no contrast agents	Time-intensive, lack of standardization	Monitoring ex vivo
MRI	Non-invasive, high diagnostic accuracy, quantification of perfusion	Reliable only with contrast, operator-dependent, false-positive enhancement by capillary leakage, availability	Monitoring in vivo
Scintigraphy	Non-invasive, early detection of perfusion issues	Nonspecific, false-positives (inflammation, osteoradionecrosis, beginning creeping substitution of new periosteal bone), inhomogeneous uptakes, limited resolution, resource- and time-intensive	Monitoring in vivo
NIRFI	Real-time, time-efficient, high resolution, sensitivity	Operator-dependent, postoperative assessment restricted to skin perfusion, slow and incomplete clearance	Harvest in vivo
Angiography	Direct visualization, measurement of vessel density, quantifiable comparison. Microangiography: precise ex vivo analysis	Invasive, no information on bone metabolism, operator-dependent, risk of thrombosis	Monitoring in vivo and ex vivo (microangiography)
Microspheres	Precise, multiple measurements without direct access	Restricted to larger capillaries, complex, isotope handling, prerequisites (thorough mixing with blood, avoid shunting and reaching a sufficient threshold of radioactivity)	Monitoring in vivo
Fluorochromes	Highly specific, quantitative measurements, dynamic studies	Fluorescence overlaps, insufficient intensity, specialized equipment and expertise, failure of deposition (inactive osteons)	Monitoring ex vivo

NIRFI, near-infrared fluorescence imaging; CT, computed tomography; MRI, magnetic resonance imaging.

**Table 4:** Recommended perfusion assessment methods according to the timeline and intended clinical or experimental use.

	Intraoperatively	Early postoperative		Late postoperative	
	Clinical use	Clinical use	Experimental use	Clinical use	Experimental use
Histology			X		X
CT		X	X (Nano-CT)	X	X (Nano-CT)
MRI		X		X	
Scintigraphy		X			
NIRFI	X				
Angiography		X	X (Microangiography)	X	X (Microangiography)
Microspheres		X		X	
Fluorochrome bone labeling			X		X

NIRFI, near-infrared fluorescence imaging; CT, computed tomography; MRI, magnetic resonance imaging.

#### Histology

Bone samples are stained with different techniques, including toluidine blue and Masson-Goldner trichrome, and assessed for the presence of osteoblasts, osteoclasts, empty lacunae, fibrous tissue, and marrow viability.^27^ Viable perfused bone presents osteoid formation and active bone remodeling. Spatial resolution remains undisputed, and functional information about bone turnover can be gained.^27^

However, it is a time-intensive examination with an incomplete three-dimensional visualization.^33^ Its postmortem performance limits its use to animal studies and renders it useless in clinical human studies where longitudinal monitoring is desirable. Small samples may not represent the entire status and interpretation may be operator-dependent.^27^ Careful interpretation is required including the effect of preparation techniques, empty lacunae found in viable bones, and viable cells found in necrotic bone up until 4 days after necrosis. Bone viability is reliably determinable only after 2 weeks.^24^

#### Nano-computed tomography

Nano-CT is an ex-vivo, non-destructive 3D imaging method derived from micro-CT, enabling sub-micrometer resolution and visualization of osteocyte lacunae. Bone may be examined without contrast agents and a morphometric analysis can be performed including vessel volume and density.^33^

Limitations include its long acquisition time, and lack of standardization. Due to motion artifacts and insufficient tube power, the technique is restricted to small ex vivo analyses.^33^ Even with nano-CT, intraosseous vessels may be missed and analysis of these is resource and time intensive.^20^

#### Magnetic resonance imaging

Gadolinium-enhanced MRI allows for dynamic examinations. Perfusion can be analyzed by time-signal intensity curves (TICs) and quantified as percent signal enhancement.^34^

Advantages include its non-invasive, repeatable character and real-time results. It has a high sensitivity of 97.5%, but only a specificity of 75%. Microvasculature including capillary blood supply and permeability can be evaluated.^34^

While loss of T1 and increase of T2 signals have been associated with early revascularization, normal signals have been associated with both normal blood flow and avascular bones. In contrast-enhanced MRI, false-positive enhancement through capillary leakage is a potential pitfall. It is not widely available and results remain operator and equipment dependent.^34^

#### Technetium-based scintigraphy

As parallels have been found between the curves of calcium uptake and blood flow, Tc-based scintigraphy has been hypothesized to provide indirect means to assess perfusion.^14^^,^^24^ Based on intravenously injected Tc-99 m, the technique is performed 2–10 days postoperatively using a gamma camera for three distinct phases.^35^ The dynamic phase captures images directly after injection to assess larger vessels. The blood pool phase assesses capillaries. The delayed phase is conducted several hours after to show bone uptake and inform about metabolic activity.^36^ The uptake can be compared to regular bones and positive uptake indicates patent anastomoses.^37^

This non-invasive procedure detects perfusion issues up to 1 week postoperatively. Absence of tracer uptake correlated with later complications and VBG failure.^35^, ^36^, ^37^

Yet, tracer uptake is nonspecific and may be increased in cases of inflammation, periosteal creep, or osteoradionecrosis. Scintigraphy after 1 week postoperatively has also been shown to reflect beginning creeping substitution of newly formed periosteal bone around necrotic VBGs rather than true perfusion, thereby introducing false positive results.^37^ These have been reported at 37.5%.^15^ In cases of inhomogeneous uptakes, SPECT is recommended.^37^ Especially in the mandible region, spatial resolution is limited.^35^ The technique is resource- and time-intensive, and therefore only recommended for specific questions and complicated cases.^36^

#### Near-infrared fluorescence imaging

NIRFI is based on the fluorescent dye ICG, which is injected intravenously and visualized in real-time by a camera with near-infrared light. Perfusion can be assessed pre-harvest, post-anastomosis or post-inset. A quantitative analysis is possible with specialized software.^38^

Venous and arterial flow, and both periosteal and endosteal perfusion are assessed in a time-efficient manner. Real-time feedback may affect intraoperative decisions, such as whether partial salvage is feasible when only parts of the flap remain perfused.^38^ Furthermore, authors have pointed out its high resolution and sensitivity.^1^

However, its proper use remains operator dependent^38^ and postoperative use is very limited as assessment will be restricted to skin perfusion. The slow and incomplete clearance of ICG intraoperatively has also been pointed out.^1^

#### Angiography

Angiography is a dynamic method to visualize perfusion. Catheters are inserted into vessels of interest, contrast medium is injected, and time-controlled X-rays are performed.^39^ Microangiography offers a precise postmortem analysis using radiopaque polymers such as Microfil.^24^ Extraosseous and intraosseous vessels can be visualized and measurement of vessel density allows for comparison to physiological bone.^12^

Its invasive character prohibits multiple examinations necessary to observe dynamic changes. It does not provide information on bone metabolism,^12^ is operator-dependent and carries the risks of vein thrombosis.^39^

#### Radioactive microspheres

Microspheres are distributed with the arterial flow and stuck in capillaries because of a diameter of 15 μm. Quantifiable radioisotope levels^40^ are proportional to the region’s blood flow. Studies have shown consistently precise results for determining the regional bone blood flow.^41^

Advantages include the possibility of multiple measurements without direct bone access.^29^

Microspheres are however restricted to larger capillaries, potentially missing microvasculature. The complex technique requires catheterization, isotope handling,^40^ thorough mixing with blood, avoidance of shunting and reaching a sufficient threshold of radioactivity.^14^

#### Fluorochrome bone labeling

Fluorochromes bind to calcium at sites of active bone mineralization and intact perfusion, where they fluoresce under specific wavelengths. Most studies use sequential or combined labeling, injecting fluorochromes at different times to form distinguishable bands in bone tissue. After harvest, either conventional microscopy or high-resolution confocal laser scanning microscopy (CLSM) can be used to detect fluorochromes.^42^ Fluorochromes used include tetracycline, 2,4-bis [N,N’-di(carbomethyl)aminomethyl] fluorescein (DCAF), xylenol orange or 1,2-dihydroxyanthracinon-e-methylene-iminodiacetic acid (alizarin complexone).^24^

Fluorochrome bone labeling is highly specific, and allows quantitative and dynamic measurements. Tetracyclines have been approved for human in vivo use. CLSM distinguishes fluorochromes with similar peak wavelengths, reducing observer bias.^42^

Limitations include its destructive nature as well as fluorescence overlaps, making distinctions difficult without CLSM. CLSM requires specialized equipment and expertise.^42^ Failure of deposition when administered at an area with inactive osteons has also been reported.^24^

### Avoiding the spontaneous angiogenic response

Several authors have pointed out the need to assess perfusion without falsification due to spontaneous angiogenic response. Indeed, neoangiogenesis in canines might restore blood flow after 2.5 weeks already, underlining the need for reliable methods of blocking revascularization. Coating VBGs in cyanoacrylate or polymethyl methacrylate has been used prevent this.^27^^,^^29^ Other authors proposed to ligate the pedicle in controls to assess the effect of revascularization from the wound.^26^ In order to provide the worst possible environment for neoangiogenesis, some authors have placed VBGs in poorly perfused subcutaneous fat.^24^

### Limitations

Characteristics between studies differed significantly, including selection of animals, VBG types, time and methods of perfusion assessment and other. No analysis of the experimental outcomes of VBGs and non-VBGs is made.

## Conclusion

We provide a comprehensive analysis of the anatomical models and techniques for perfusion assessment used in VBGs. Diverse techniques are employed to examine graft viability and vascular architecture. Every perfusion assessment method was shown to have its limitations. Therefore, a multimodal approach seems to be necessary to provide a comprehensive understanding of graft perfusion and viability. The ideal composition of methods will vary depending on the respective experimental setting and exact research question.

## Funding

This research did not receive any specific grant from funding agencies in the public, commercial, or not-for-profit sectors.

## Ethical approval

Not required. Data extracted from included studies and used for analyses can be made available upon request but is not publicly available.

## Declaration of competing interest

None.
